# Antiphospholipid antibodies and anticoagulant therapy: capillaroscopic findings

**DOI:** 10.1186/s13075-021-02551-6

**Published:** 2021-06-27

**Authors:** Giorgia Ferrari, Emanuele Gotelli, Sabrina Paolino, Giampaola Pesce, Luca Nanni, Barbara Maria Colombo, Greta Pacini, Carlotta Schenone, Carmen Pizzorni, Alberto Sulli, Vanessa Smith, Maurizio Cutolo

**Affiliations:** 1 Laboratory of Experimental Rheumatology and Academic Division of Clinical Rheumatology, Department of Internal Medicine and Specialties (DiMI), University of Genova, IRCCS San Martino Polyclinic, Viale Benedetto XV, n° 6 - 16132 Genoa, Italy; 2 Autoimmunity Laboratory, Department of Internal Medicine and Specialties (DiMI), University of Genova, IRCCS San Martino Polyclinic, Genoa, Italy; 3 Medicine Laboratory, IRCCS San Martino Polyclinic, Genoa, Italy; 4 IRCCS San Martino Polyclinic, Genova – Italian Cardiovascular Network, Genoa, Italy; 5 Department of Rheumatology, Ghent University Hospital – Department of Internal Medicine, Ghent University, Ghent, Belgium; 6 Unit for Molecular Immunology and Inflammation, VIB Inflammation Research Center (IRC), Ghent, Belgium

**Keywords:** Nailfold capillaroscopy, Antiphospholipid syndrome, Connective tissue diseases, Systemic sclerosis, Systemic lupus erythematosus, Anticoagulant therapy

## Abstract

**Background:**

Antiphospholipid syndrome (APS) is a systemic autoimmune disease characterized by specific vascular and obstetric manifestations and by antiphospholipid antibodies (aPL) positivity. Microvascular damage in the course of APS and “aPL carrier” patients without symptoms is poorly investigated.

**Objectives:**

This study aims to compare nailfold videocapillaroscopy (NVC) microvascular parameters in APS patients and non-symptomatic "aPL carriers" and to investigate their possible correlations with different aPL subtypes.

**Methods:**

NVC was performed during standard evaluations in 18 APS patients (mean age 50 ± 13.8 years), 24 "aPL carriers" without symptoms (mean age 46.4 ± 16.4 years), and 18 control patients (CTR) (mean age 74 ± 12.5 years) taking oral anticoagulants for non-immunological indications (i.e., cardiovascular accidents). All patients were investigated for the presence of dilated capillaries, giant capillaries, microhemorrhages, capillary loss, and further non-specific/specific abnormalities (i.e., branched “bushy” capillaries, sign of neoangiogenesis) by NVC. Every alteration was also classified according to a semi-quantitative score. Lupus anticoagulant, anticardiolipin antibodies, and antibeta2 glycoprotein I antibodies were tested in each patient.

**Results:**

APS patients showed at NVC increased frequency of microhemorrhages (*p* = 0.039)—particularly a “comb-like” pattern (parallel hemorrhages) (*p* = 0.002)—than "aPL carriers". Of note, there were no significant differences concerning the isolated number of microhemorrhages between APS and the CTR group (*p* = 0.314), but “comb-like” hemorrhages were significantly more frequent in the APS group (*p* = 0.034). Not any significant correlation was found between the aPL subtypes and NVC parameters.

**Conclusions:**

APS patients showed significantly a greater number of non-specific NVC abnormalities than "aPL carriers", particularly the “comb-like” NVC pattern. Oral anticoagulants may represent a confounding factor for isolated microhemorrhages. Not any correlation was found between aPL subtypes and NVC parameters. Further investigations are needed to better characterize the microvascular endothelium damage induced by aPL.

## Introduction

Antiphospholipid syndrome (APS) is a systemic autoimmune disease characterized by arterial and/or venous thrombosis and/or obstetric morbidity, associated with the presence in the serum of specific autoantibodies, called antiphospholipid antibodies (aPL) [1]. The aPL used as diagnostic criteria for APS include anticardiolipin antibodies (aCL), antibeta2 glycoprotein I antibodies (anti-b2GPI), and lupus anticoagulant (LAC) [2]. A single, double, or triple aPL positivity of an individual is defined as “aPL profile” and predicts the onset of macrovascular thrombosis as well as may influence the treatment strategy. Importantly, subjects with isolated detection of aPL in absence of clinical thrombotic manifestations cannot be classified as APS patients, but only as “aPL carriers” [3, 4].

To date, APS microangiopathy is poorly characterized. Nailfold videocapillaroscopy (NVC) has been performed to assess microcirculation in these patients and the most reported alterations are capillary tortuosity and microhemorrhages [5].

**Fig. 1:**
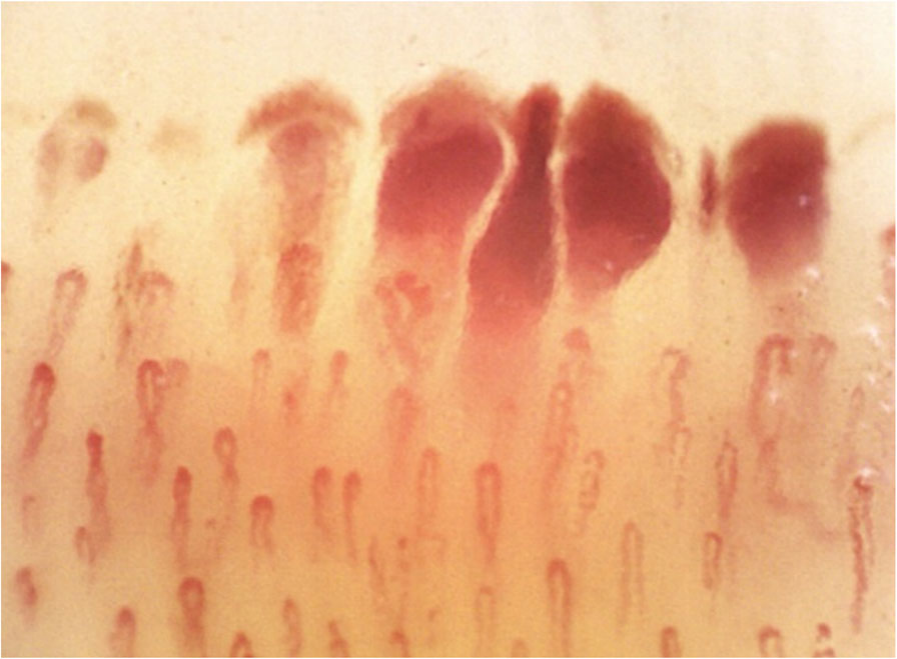
Example of “comb-like” recent microhemorrhages in APS patient. Nailfold videocapillaroscopy (magnification × 200). APS: antiphospholipid syndrome

Multiple hemorrhages from normal shaped capillaries, which appear parallel/linear and arranged perpendicularly to the nailfold bed, are called “comb-like” hemorrhages and are suggestive of APS (Fig. 1) [6]. However, the correlation of these NVC non-specific abnormalities with specific aPL subtypes has always been contrasting [7, 8]. Moreover, not any data about NVC abnormalities in "aPL carriers" is available to date.

Therefore, the aim of this study was the characterization of the microvascular damage in APS patients and "aPL carriers", investigating possible correlations between NVC microvascular parameters and different aPL subtypes. A control group of patients (CTR) on regular warfarin therapy for cardiovascular indications without aPL positivity has been also taken into account, in order to exclude possible NVC alterations due to anticoagulant therapy.

## Methods

### Study population

Eighteen APS patients (mean age 50 ± 13.8 SD years, 13 females and 5 males), twenty-four "aPL carriers" (mean age 46.4 ± 16.4 SD years, 23 females and 1 male), and eighteen CTR patients (mean age 74 ± 12.5 SD years, 8 females and 10 males) underwent NVC evaluation during their standard evaluations at the Rheumatology Department of Genoa University (Italy).

The complete medical history (including episodes of vascular thrombosis, pregnancy-related morbidity, anticoagulant therapy) and laboratory findings of enrolled patients were available in their clinical files.

APS diagnosis was made according to revised 2006 Sapporo classification criteria [2]. APS patients had either a primary syndrome or a secondary APS related to systemic lupus erythematosus (SLE—2019 EULAR/ACR criteria) [9]. Patients with an established diagnosis of systemic sclerosis (SSc), Sjögren’s syndrome (SjS), mixed connective tissue disease (MCTD), and idiopathic inflammatory myopathy (IIM) were excluded from the study [10–13].

CTR patients were on warfarin therapy for either atrial fibrillation (stable international normalized ratio (INR) from 2 to 3), mechanical heart valve implant (stable INR from 2.5 to 3.5), and/or deep venous thrombosis (stable INR from 2 to 3), and they had a negative aPL profile. Exclusion criteria of CTR patients were connective tissue diseases (CTDs), Raynaud’s phenomenon, and recent traumatic events to their hands.

Clinical and immunological parameters of enrolled patients are summarized in Tables 1 and 2.

**Table 1:** Clinical and immunological characteristics of APS patients, "aPL carriers", and CTR patients

	APS	"aPL carriers"	CTR
**Total patients**	18	24	18
**Female–male sex**	13–5	23–1	8–10
**Age (years, mean ± SD)**	50 ± 13.8	46.4 ± 16.4	74 ± 12.5
**Disease duration (years, mean ± SD)**	7.6 ± 7.7	–	–
**Primary–secondary forms**	12–6	17–7	–
**Arterial/venous thrombosis**	16/18 (88.9)	–	9 (50.0)
**Pregnancy morbidity**	3/13 (23.1)	–	–
**Atrial fibrillation**	–	–	6 (33.3)
**Mechanical heart valves**	–	–	3 (16.7)
**Arterial hypertension**	6	8	6
**Diabetes mellitus**	0	1	2
**LAC positivity**	5/18 (27.8)	5/24 (20.8)	–
**ACL IgG positivity**	4/18 (22.2)	9/24 (37.5)	–
**ACL IgM positivity**	9/18 (50.0)	5/24 (20.8)	–
**Anti-b2GPI IgG positivity**	5/18 (27.8)	8/24 (33.3)	–
**Anti-b2GPI IgM positivity**	8/18 (44.4)	12/24 (50.0)	–
**Single positivity**^**a**^	10/18 (55.5)	13/24 (54.1)	–
**Double positivity**^**b**^	5/18 (27.8)	10/24 (41.7)	–
**Triple positivity**^**c**^	3/18 (16.7)	1/24 (4.2)	–

*APS* antiphospholipid syndrome, *aPL* antiphospholipid antibodies, *CTR* control, *SD* standard deviation, *LAC* lupus anticoagulant, *ACL* anticardiolipin antibodies, *anti-b2GPI* antibeta2 glycoprotein I antibodies

^a^Single positivity is defined as the positivity of only one between LAC, ACL IgG/M, and anti-b2GPI IgG/M

^b^Double positivity is defined as the positivity of two between LAC, ACL IgG/M, and anti-b2GPI IgG/M, variously mixed

^c^Triple positivity is defined as the positivity of three between LAC, ACL IgG/M, and anti-b2GPI IgG/M, variously mixed

**Table 2:** Clinical and immunological characteristics of SLE-related APS patients and SLE-"aPL carriers"

	SLE-APS	SLE-"aPL carriers"
**Total patients**	6	7
**Female–male sex**	6–0	7–0
**Age (years, mean ± SD)**	53 ± 12	46 ± 12
**Disease duration (years, mean ± SD)**	18 ± 15	15 ± 11
**Remission (SLEDAI < 2)**	6	7
**Active disease (SLEDAI > 2)**	0	0
**Clinical domains**		
***Cutaneous domain***	5	7
***Arthritis domain***	1	2
***Neurological domain***	1	0
***Serositis domain***	1	1
***Hematologic domain***	1	3
***Renal domain***	0	1
**Current therapy**		
***Glucocorticoids (prednisone ≤ 5 mg per day)***	5	7
***Hydroxychloroquine***	2	5
***cDMARDs (methotrexate, azathioprine, mycophenolate mofetil)***	4	4

*SLE* systemic lupus erythematosus, *SLEDAI* systemic lupus erythematosus disease activity index, *APS* antiphospholipid syndrome, *aPL* antiphospholipid antibodies, *SD* standard deviation, *cDMARDs* conventional disease-modifying anti-rheumatic drugs

This retrospective study was conducted in accordance with the principles of the Declaration of Helsinki and Good Clinical Practice. All the patients signed the mandatory written informed consent to manage their clinical data according to the rules of the Hospital/University at the time of their first visit in the Clinic.

### Antiphospholipid antibodies detection

ACL and anti-b2GPI antibodies of IgG and/or IgM isotype have been measured by a standardized ELISA, according to recommended procedures (PerkinElmer, Euroimmun, MA, USA). They were considered positive if present in serum or plasma, in a medium or high titer (either > 40 IgM or IgG phospholipid units or > 99^th^ centile), on two or more occasions, at least 12 weeks apart.

LAC was detected according to the International Society on Thrombosis and Hemostasis guidelines and considered positive if present in the plasma, on two or more occasions, at least 12 weeks apart [2].

### Nailfold videocapillaroscopy

NVC has been performed by the same physician (CP) using an optical probe with a × 200 magnification lens connected to picture analysis software (Videocap, DS Medica, Milan, Italy).

According to the standardized procedures, each patient remained in the test room for a minimum of 15 min before the NVC at the temperature of 20–22 °C. Two pictures of the 2-mm area in the middle of the nailfold bed of all the fingers, thumbs excluded, have been collected for each subject [14]. The following capillaroscopic parameters have been assessed: normal capillaries (including non-specific abnormalities: hairpin-shaped, tortuous, or crossing capillaries with branch diameters < 20 μm), dilated capillaries (irregular or homogeneous increase of capillary diameter between 20 and 50 μm), giant capillaries (homogeneously dilated normal shaped loops with a diameter ≥ 50 μm), microhemorrhages (dark masses attributable to hemosiderin deposit) with particular attention to “comb-like” hemorrhages, abnormal shapes (i.e., branched “bushy” capillaries, sign of neoangiogenesis), and capillary number reduction. A validated semi-quantitative rating scale has been adopted to score each NVC capillary abnormality detected (0, no changes; 1, < 33% of capillary alterations/reduction; 2, 33–66% of capillary alterations/reduction; 3, > 66% of capillary alterations/reduction per linear millimeter) [15, 16].

### Statistical analysis

Continuous variables were reported as medians and interquartile range (IQR) or standard deviation (SD) when appropriate, while categorical variables as count and percentage. Normality of distribution of continuous variables was to be assessed by visual inspection. The chi-square test or Fisher’s exact test was be used to compare categorical variables while the Mann-Whitney test was used to compare continuous variables. Any *p* values equal or lower than 0.05 was considered statistically significant.

## Results

### NVC findings

In the APS cohort, 3 patients showed a normal NVC pattern, while 15 patients showed non-specific abnormalities. In the "aPL carriers" cohort, 8 patients showed a normal NVC pattern and 16 patients showed non-specific abnormalities. In the CTR group, 7 patients showed a normal NVC pattern and 11 patients showed non-specific NVC abnormalities. The different NVC findings are presented in Table 3.

**Table 3:** Comparison of NVC alterations between APS and "aPL carriers" and between APS and CTR group

VCP evaluation	APS	"aPL carriers"	*p*-value	CTR	*p*-value
Global pattern (%)					
Normal	3 (16.7)	8 (33.3)	0.299	7 (38.9)	0.264
Non-specific alterations	15 (83.3)	16 (66.7)	0.299	11 (61.1)	0.264
AB score, median [IQR]					
A-score	2.0 [2.0, 3.0]	1.0 [1.0, 2.0]	0.004	1.5 [1.0, 2.0]	0.021
B-score	0.0 [0.0, 0.8]	0.0 [0.0, 0.0]	0.098	0.0 [0.0, 0.0]	0.674
Dilated capillaries					
Median score [IQR]	1.0 [1.0, 2.0]	1.0 [1.0, 1.0]	0.009	1.0 [1.0, 1.0]	0.027
Number of patients (%)	17 (94.4)	20 (83.3)	0.536	18 (100.0)	1
Giant capillaries					
Number of patients (%)	0 (0.0)	0 (0.0)	–	0 (0.0)	–
Microhemorrhages					
Median score [IQR]	1.0 [0.0, 1.0]	0.0 [0.0, 1.0]	0.034	0.0 [0.0, 1.0]	0.186
Number of patients (%)	12 (66.7)	8 (33.3)	0.032	8 (44.4)	0.314
Abnormal shapes					
No. of patients (%)	2 (11.1)	0 (0.0)	0.178	2 (11.1)	1
Reduced numerosity					
No. of patients (%)	1 (5.6)	0 (0.0)	0.429	0 (0.0)	1
Absolute value of capillaries, median [IQR]	9.5 [9.0, 10.0]	10.0 [9.0, 10.0]	0.946	10.5 [10.0, 11.8]	0.010
Single hemorrhages					
Median [IQR]	0.0 [0.0, 0.8]	0.0 [0.0, 0.2]	0.767	0.0 [0.0, 1.0]	0.461
No. of patients (%)	5 (27.8)	6 (25.0)	0.875	7 (38.9)	0.72
“Comb-like” hemorrhages					
Median [IQR]	0.5 [0.0, 1.0]	0.0 [0.0, 0.0]	0.007	0.0 [0.0, 0.0]	0.029
No. of patients (%)	8 (50)	3 (12.5)	0.020	3 (16.7)	0.034

*APS* antiphospholipid syndrome, *aPL* antiphospholipid antibodies, *CTR* control, *IQR* interquartile range

The number of patients with dilated capillaries was not statistically different in the three groups (APS vs "aPL carriers", *p* = 0.536 and APS vs CNT, *p* = 1.000, respectively), but the semi-quantitative score was significantly higher in the APS group (APS vs "aPL carriers", *p* = 0.009 and APS vs CNT, *p* = 0.002). Microhemorrhages were significantly more frequent in APS patients than in "aPL carriers" (*p* = 0.032), whereas not any significant difference was found between APS patients and the CTR group (*p* = 0.314). However, “comb-like” hemorrhages (NVC pattern) were statistically more represented in the APS group (than "aPL carriers" *p* = 0.003, and compared with CTR *p* = 0.01).

**Fig. 2:**
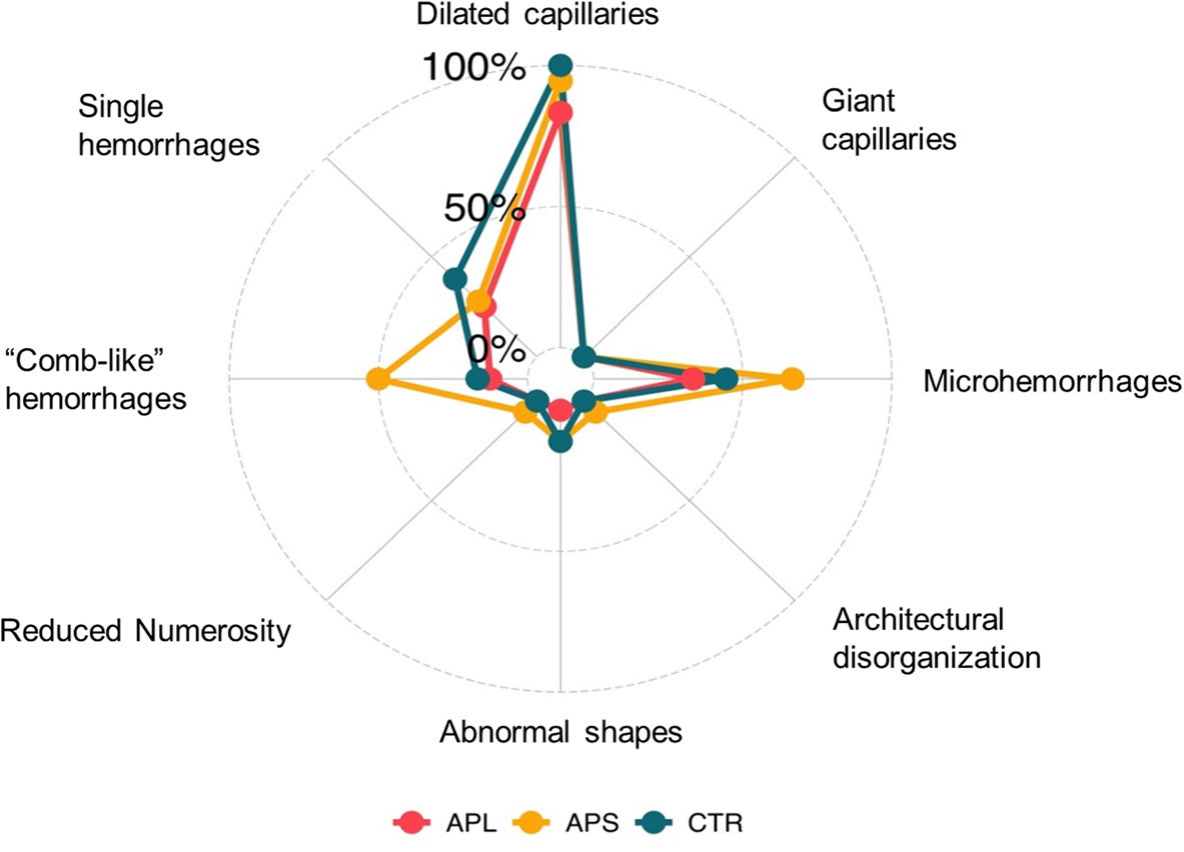
Radar plot of NVC alterations. For each variable, the proportion of patients of each group with that characteristic (items’ score > 0) is reported. The groups are represented with different colors. APS: antiphospholipid syndrome, aPL: antiphospholipid antibodies carriers, CTR: control group

Among the entire cohort of patients, none showed giant capillaries at NVC, whereas a lower capillary density was detected in just one APS patient. Non-specific abnormal capillaries were observed only in APS patients (11.1%) and CTR groups (11.1%). A “radar” plot reporting all NVC variables is provided in Fig. 2.

### NVC alterations and immunological profile

Not any significant correlation was found between the immunological profile and non-specific nailfold microvascular array abnormalities—notably, microhemorrhages, “comb-like” hemorrhages, abnormally shaped capillaries, capillary density—or specific aPL isotypes (Table 4).

**Table 4:** NVC alterations in APS and "aPL carriers" groups and their correlation with aPL isotypes

	Microhemorrhages	***p***-value	"Comb-like"	***p***-value
**Total number**	20		12	
**Any positive IgG (%)**	8 (40.0)	0.906	3 (25.0)	0.526
**Any positive IgM (%)**	13 (65.0)	0.845	9 (75.0)	0.405
**Any positive anti-b2GPI (%)**	16 (80.0)	0.541	9 (75.0)	1
**Any positive ACL (%)**	12 (60.0)	1	7 (58.3)	1
**Positive ACL IgG (%)**	7 (35.0)	0.915	3 (25.0)	0.822
**Positive ACL IgM (%)**	8 (40.0)	0.659	6 (50.0)	0.31
**Positive anti-b2GPI IgG (%)**	7 (35.0)	0.915	3 (25.0)	0.822
**Positive anti-b2GPI IgM (%)**	12 (60.0)	0.276	8 (66.7)	0.258
**Positive LAC (%)**	4 (20.0)	0.783	3 (25.0)	1

*ACL: anticardiolipin antibodies, anti-b2GPI: antibeta2 glycoprotein I antibodies, LAC: lupus anticoagulant*

## Discussion

This study compares NVC microvascular findings in a cohort of APS patients and "aPL carriers" for the first time, with a control group of patients in chronic therapy with warfarin for non-immunological indications.

NVC is to date the best tool to analyze microvascular abnormalities in CTDs [17, 18]. Additionally, NVC alterations in course of SSc are well-defined and correlate with organ involvement and disease progression, in contrast to other CTDs—such as in SLE, SjS, MCTD, and IIM—in which NVC alteration is less disease-specific [19–24].

In this investigation, both the primitive and the SLE-secondary forms of APS were considered, excluding association with SSc, IIM, and MCTD due to the presence of already defined NVC patterns (scleroderma spectrum disorders).

In 2018, the European Alliance of Associations for Rheumatology (EULAR) study group on Microcirculation in Rheumatic Diseases reported a significantly higher prevalence of tortuous capillaries, abnormal morphology, and hemorrhages in SLE patients than in healthy controls [23]. However, in our study, SLE patients were equally distributed between the APS and "aPL carriers" cohorts, thus not representing a bias for the statistical analysis.

As previously observed by other authors, APS patients showed a large variety of non-specific NVC abnormalities [25]. Particularly, microhemorrhages have been classically associated with the diagnosis of full-blown APS [8]. As a matter of fact, in our study, isolated microhemorrhages were observed in APS patients, "aPL carriers", and CTR; however, only APS patients showed a significantly higher frequency of “comb-like” parallel hemorrhages at NVC.

Bernardino et al. very recently reported the lack of association between NVC microhemorrhages in APS patients and ongoing anticoagulant treatment. Divergently, we have reported in this study a high incidence of isolated microhemorrhages in CTR patients who received a stable treatment with warfarin [26]. Hence, anticoagulant therapies might be a confounding factor for isolated microhemorrhages, but they do not appear to be associated with the NVC “comb-like” pattern. Thus, it can be suggested, also according to what previously reported by Bernardino et al., that NVC “comb-like” pattern may be a particular NVC marker in APS patients [26].

Microhemorrhages detected by NVC are relevant also in other CTDs, such as SSc, in which they are a good indicator of the steady-state level of disease activity [27, 28].

Additionally, the lack of significance of NVC alterations in "aPL carriers" may reinforce the so-called two hits hypothesis for the APS pathogenesis. Indeed, an isolated aPL positivity is not sufficient to produce an endothelium injury leading to an overt vascular thrombosis that only occurs when a second triggering factor intervenes [29, 30]**.**

Regarding the correlations between NVC alterations and specific aPL positivity, Bongard et al. have described more capillary abnormalities in ACL-positive than aCL-negative SLE patients, while Bernardino et al. have reported a significant association between ACL and the absence of hemorrhage in APS patients [26, 31]. Additionally, Pyrpasopoulou et al. have reported a significant correlation between NVC microhemorrhages and APS clinical manifestations, still not any significant association with aPL subtypes was described [8, 32]. Finally, a previous study detected a higher incidence of capillary hemorrhages and hemosiderin deposits in aPL IgG^+^-IgM^+^ patients, regardless of specific aCL or anti-b2GPI positivity [7].

Moving from this background, the present study aimed to investigate potential correlations between NVC alterations and different aPL subtypes in APS patients and "aPL carriers". Still, our analysis failed to find any statistically significant correlation between these parameters because of the small sample size.

The present study has some limitations.

Firstly, being a monocentric study, the sample of enrolled patients was numerically limited. Additionally, in the APS cohort, obstetric patients are under-represented—being only a quarter of the group—thus possibly affecting the results’ interpretation. It should also be mentioned that CTR had, on average, a higher age than APS patients and "aPL carriers" as well as a strong male prevalence. The age difference could reasonably be explained by the increased clinical indication for anticoagulant therapies in older age. Still, microhemorrhages are usually not reported in elderly patients, reinforcing the suspicion of anticoagulant therapy interference [8]. The strong male prevalence in the CTR group is due to the gender-related difference in the incidence of cardiovascular disease (which is the first indication for anticoagulant therapy) [33].

In conclusion, this study highlights significant NVC differences between APS and "aPL carriers" (e.g., “comb-like” hemorrhages), probably reflecting different steps in the pathogenesis of the full-blown disease. Microvascular endothelial injury could be more thoroughly identified by nailfold biopsies in both conditions, however, always taking into account the mechanical endothelial stress in course of anticoagulant therapy.

In fact, anticoagulants might be a confounding factor for the presence of isolated microhemorrhages, this latter aspect deserving further investigation in larger cohorts.

However, NVC “comb-like” hemorrhages appear to be a specific marker of APS, regardless of the concomitant anticoagulant therapy. Interestingly, it is still unclear whether the presence of these specific hemorrhages is an epiphenomenon of concomitant endothelial damage or may instead play a predictive role for other vascular complications.

Additional studies on larger cohorts of patients are needed to further characterize these preliminary data on NVC abnormalities in APS patients and "aPL carriers".

## Data Availability

The authors state they have full control of all primary data and agree to allow the journal to review their data after reasonable request.

## Abbreviations

APS: Antiphospholipid syndrome
NVC: Nailfold videocapillaroscopy
CTR: Control patients
aPL: Antiphospholipid antibodies
aCL: Anticardiolipin antibodies
anti-b2GPI: Antibeta2 glycoprotein I antibodies
LAC: Lupus anticoagulant
SLE: Systemic lupus erythematosus
SSc: Systemic sclerosis
SjS: Sjögren’s syndrome
MCTD: Mixed connective tissue disease
IIM: Idiopathic inflammatory myopathy
IQR: Interquartile range
SD: Standard deviation
CTDs: Connective tissue diseases
EULAR: European Alliance of Associations for Rheumatology
